# Correlation Between Postoperative Complications and Serum Albumin Levels in Abdominal Surgery: A Prospective Observational Study

**DOI:** 10.7759/cureus.86508

**Published:** 2025-06-21

**Authors:** Sumeet Kumar, Kanchan Sonelal Baitha, Anand Dev, Prem Prakash, Gagan Gunjan

**Affiliations:** 1 General Surgery (Trauma and Emergency), Indira Gandhi Institute of Medical Sciences, Patna, IND; 2 General Surgery, Indira Gandhi Institute of Medical Sciences, Patna, IND; 3 Critical Care Medicine, Indira Gandhi Institute of Medical Sciences, Patna, IND; 4 General Medicine, Rajendra Institute of Medical Sciences, Ranchi, IND

**Keywords:** abdominal surgery, hypoalbuminemia, nutritional biomarker, postoperative complications, serum albumin, surgical outcomes

## Abstract

Background

Hypoalbuminemia is a widely recognized marker of malnutrition and systemic inflammation. Its association with postoperative complications is increasingly emphasized in surgical risk stratification, particularly in abdominal procedures. However, limited prospective data from Indian populations exist to validate its predictive utility in routine clinical practice.

Objective

This study aimed yo assess the correlation between preoperative serum albumin levels and the incidence of postoperative complications in patients undergoing abdominal surgery.

Methods

This prospective observational study was conducted over a nine-month period at Indira Gandhi Institute of Medical Sciences (IGIMS), Patna. A total of 69 patients undergoing elective or emergency abdominal surgery were enrolled. Preoperative serum albumin levels were measured within 24 hours prior to surgery, and patients were categorized into hypoalbuminemic (<3.5 g/dL) and normoalbuminemic (≥3.5 g/dL) groups. Postoperative complications, duration of hospital stay, and relevant demographic and clinical variables were recorded. Statistical analyses included Chi-square tests, Mann-Whitney U tests, and multivariate logistic regression.

Results

Among 69 patients, 45 (65.2%) were hypoalbuminemic. Postoperative complications were significantly more frequent in the hypoalbuminemic group, with 26 patients (57.8%) experiencing complications compared to only three of 24 patients (12.5%) in the normoalbuminemic group (p < 0.001). The mean hospital stay was also significantly longer in hypoalbuminemic patients (10.1 ± 2.9 days vs. 6.9 ± 2.0 days; p < 0.0001; Cohen’s d = 1.32). Multivariate logistic regression identified hypoalbuminemia (OR = 12.68; 95% CI: 3.01-53.53; p = 0.001) and surgery duration (p = 0.045) as independent predictors of postoperative complications, while age and comorbidity status were not statistically significant.

Conclusion

Preoperative hypoalbuminemia is a strong and independent predictor of postoperative complications and prolonged hospitalization in patients undergoing abdominal surgery. Serum albumin should be routinely evaluated as part of preoperative assessment to guide perioperative planning and optimize outcomes.

## Introduction

Abdominal surgery, encompassing both elective and emergency procedures, remains a cornerstone of surgical practice in India. Despite advancements in operative techniques and perioperative care, postoperative complications continue to pose significant challenges, contributing to prolonged hospitalization, increased healthcare costs, and elevated morbidity and mortality. A variety of factors influence surgical outcomes, including the patient’s age, nutritional status, preexisting comorbidities, and intraoperative variables. Among these, nutritional status has gained increasing attention, with serum albumin emerging as a valuable biomarker in the risk stratification of surgical patients [1,2].

Serum albumin, the most abundant plasma protein synthesized by the liver, accounts for approximately 50-60% of total serum protein. It plays multiple physiological roles, including maintenance of oncotic pressure, transport of endogenous and exogenous substances, and modulation of inflammatory responses [3]. Importantly, albumin is classified as a negative acute-phase reactant, and its levels decline in response to systemic inflammation, infection, or surgical stress. In acute surgical settings, hypoalbuminemia may develop due to a combination of capillary leakage, hemodilution, and altered hepatic synthesis. These mechanisms differ from chronic conditions, where malnutrition, protein-losing enteropathy, or nephropathy may predominate [4].

In abdominal surgery, hypoalbuminemia has been consistently associated with adverse postoperative outcomes, including surgical site infections (SSIs), delayed wound healing, anastomotic leakage, wound dehiscence and increased reoperation rates. Albumin not only reflects a patient’s nutritional reserves but also correlates with immune competence and the ability to mount an effective response to surgical stress. As such, it is increasingly used as a surrogate marker for physiological resilience and recovery potential [5].

Several studies have underscored the predictive value of preoperative serum albumin levels. Gibbs et al. [1] identified hypoalbuminemia as a strong independent predictor of morbidity and mortality in general surgery patients. Similarly, Gupta et al. [2] demonstrated that patients with low serum albumin undergoing gastrointestinal surgery were at significantly higher risk for postoperative complications. Other investigations, including those by Martin et al. [6] and Adogwa et al. [7], have extended these observations to patients undergoing colorectal and spinal surgeries, respectively, highlighting albumin’s relevance across surgical disciplines.

Preoperative nutritional optimization has gained recognition as a pivotal aspect of patient preparation for surgery, particularly in abdominal procedures where postoperative outcomes are closely linked to baseline physiological status. Increasingly, clinical frameworks incorporate nutritional assessment tools to better stratify risk and improve perioperative care. In addition, studies such as those by Kudsk et al. have demonstrated that both albumin levels and surgical wound class can jointly predict the likelihood of postoperative complications [8].

Given the burden of surgical complications and the resource constraints in many Indian healthcare settings, early identification of at-risk patients is crucial. Serum albumin offers a cost-effective, readily available marker that may inform perioperative decision-making and facilitate targeted interventions. This study was therefore designed to investigate the correlation between serum albumin levels and postoperative complications in patients undergoing abdominal surgery, with the aim of validating its utility as a predictive biomarker in clinical practice.

## Materials and methods

This prospective observational study was designed to examine the correlation between preoperative serum albumin levels and the incidence of postoperative complications in patients undergoing abdominal surgery. The primary objective was to evaluate the relationship between hypoalbuminemia and the development of adverse outcomes such as surgical site infections, anastomotic leakage, wound dehiscence and delayed wound healing. Secondary objectives included assessing the predictive utility of serum albumin for identifying high-risk patients, and analyzing the influence of nutritional status on surgical recovery and hospital course. The study was conducted over a nine-month period from September 2024 to May 2025 at the Indira Gandhi Institute of Medical Sciences (IGIMS), Sheikhpura, Patna, Bihar.

A total of 69 patients undergoing abdominal surgery were enrolled in the study. The sample size was calculated using the formula n = z² × p (1 − p) / ε², assuming a 90% confidence level and a 10% margin of error. Consecutive consenting patients aged between 18 and 70 years were included. Exclusion criteria comprised patients older than 70 years, individuals with known malignancy, cachexia, or immunocompromised status (such as HIV or long-term steroid therapy), and those who declined consent.

Serum albumin was used as the key variable of interest. Patients with a serum albumin level equal to or greater than 3.5 g/dL were considered to have adequate protein reserves, while levels below 3.5 g/dL indicated hypoalbuminemia, suggestive of malnutrition in stable, well-hydrated individuals. Preoperative serum albumin was measured within 24 hours prior to surgery using an automated clinical chemistry analyzer.

Clinical data were collected using a standardized proforma and included demographic information (age, sex), comorbidities, type and duration of surgery, and intraoperative and postoperative parameters. Postoperative complications, such as surgical site infections, anastomotic breakdown, wound dehiscence and delayed wound healing, were monitored daily until discharge or the 30th postoperative day, whichever occurred first. Complications were documented according to predefined diagnostic criteria based on clinical signs, laboratory results, and radiological findings,

Data were entered into Microsoft Excel (Microsoft Corporation, Redmond, WA, USA) and analyzed using IBM SPSS Statistics for Windows, Version 26.0 (released 2018, IBM Corp., Armonk, NY). Continuous variables were summarized as means with standard deviations or medians with interquartile ranges, depending on normality. Categorical variables were expressed as counts and percentages. Comparisons between groups were performed using the Chi-square test for categorical variables, and the Mann-Whitney U test for continuous variables, as appropriate. A multivariate logistic regression analysis was conducted to adjust for potential confounding variables, including age, comorbidities, and surgical factors. A p-value of less than 0.05 was considered statistically significant.

All ethical guidelines were followed in accordance with the Declaration of Helsinki. Ethical clearance was taken from the Institutional Ethical Committee of IGIMS (ethical clearance number 146/IEC/IGIMS/2024, dated 04/09/2024). Written informed consent was obtained from each participant prior to enrollment. Confidentiality was ensured throughout the study, with all datasets anonymized before statistical analysis. Patients were informed that participation was voluntary and that refusal to participate would not impact their clinical care.

## Results

Baseline characteristics of the study population

A total of 69 patients undergoing abdominal surgery were included in the study. The mean age was 50.7 ± 9.0 years. The majority were male (n = 44, 63.8%), and just over half underwent emergency procedures (n = 35, 50.7%). Comorbidities were common, with diabetes in 15 patients (21.7%), hypertension in 13 (18.8%), and anemia in 10 (14.5%). Multiple comorbidities were present in nine patients (13.0%), while 16 patients (23.2%) had no documented comorbidities. Serum albumin levels measured within 24 hours prior to surgery showed that 45 patients (65.2%) were hypoalbuminemic (<3.5 g/dL), while 24 patients (34.8%) had normal levels (≥3.5 g/dL). These characteristics are summarized in Table 1.

**Table 1:** Baseline characteristics of the study population

Variable	n (%)
Age (mean ± SD)	50.7 ± 9.0
Gender	
Male	44 (63.8%)
Female	25 (36.2%)
Type of surgery	
Elective	34 (49.3%)
Emergency	35 (50.7%)
Comorbidities	
None	16 (23.2%)
Diabetes	15 (21.7%)
Hypertension	13 (18.8%)
Anemia	10 (14.5%)
Multiple	9 (13.0%)
Serum albumin levels	
<3.5 g/dL	45 (65.2%)
≥3.5 g/dL	24 (34.8%)

Prevalence of postoperative complications

Among the 69 patients included in the study, 29 (42.0%) experienced at least one postoperative complication. The complication rate was notably higher in the hypoalbuminemic group, with 26 of 45 patients (57.8%) in the <3.5 g/dL category developing complications, compared to only 3 of 24 patients (12.5%) in the ≥3.5 g/dL group. These findings are summarized in Table 2.

**Table 2:** Prevalence of postoperative complications by albumin category

Albumin category	Complication: Yes	Complication: No
<3.5 g/dL	26 (59.0%)	18 (41.0%)
≥3.5 g/dL	3 (12.0%)	22 (88.0%)
Total	29 (42.0%)	40 (58.0%)

Association between serum albumin and postoperative complications

To assess the association between serum albumin levels and postoperative complications, a Chi-square test of independence was performed. The results revealed a statistically significant relationship between hypoalbuminemia (<3.5 g/dL) and the occurrence of postoperative complications (χ² = 12.64, df = 1, p = 0.0004), indicating that patients with lower preoperative albumin levels were more likely to experience adverse outcomes. Cramer’s V was calculated at 0.43, suggesting a moderate strength of association between serum albumin status and postoperative morbidity. A detailed summary of the statistical test is provided in Table 3.

**Table 3:** Statistical test summary for the albumin level and complication risk

Test performed	Chi-square test of independence
Chi-square value (χ²)	12.64
Degrees of freedom (df)	1
p-value	0.0004
Cramer’s V	0.43 (moderate association)

Comparison of hospital stay duration by albumin status

A comparison of hospital stay duration revealed a statistically significant difference between the two groups. Patients with preoperative hypoalbuminemia (<3.5 g/dL) had a mean hospital stay of 10.1 ± 2.9 days, while those with normal serum albumin levels (≥3.5 g/dL) had a shorter mean duration of 6.9 ± 2.0 days. The difference in hospital stay duration was statistically significant (Mann-Whitney U = 898.50, p < 0.0001), with a Cohen’s d of 1.32, indicating a large effect size. These findings suggest that hypoalbuminemia is associated with prolonged hospitalization following abdominal surgery. A summary of the statistical comparison is provided in Table 4.

**Table 4:** Hospital stay duration by albumin status

Hypoalbuminemia group (mean ± SD)	10.1 ± 2.9 days
Normal albumin group (mean ± SD)	6.9 ± 2.0 days
Test performed	Mann–Whitney U test
Mann–Whitney U	898.50
p-value	< 0.0001
Cohen’s d	1.32 (large effect size)

Multivariate logistic regression analysis

A multivariate logistic regression model was constructed to assess the independent predictors of postoperative complications, adjusting for serum albumin level, age, comorbidity status, and surgery duration. Hypoalbuminemia (<3.5 g/dL) emerged as a strong independent predictor of postoperative complications, with an odds ratio (OR) of 12.68 (95% CI: 3.01-53.53, p = 0.001). Surgery duration was also significantly associated with increased risk (OR = 1.02 per minute, 95% CI: 1.00-1.04, p = 0.045). In contrast, age and comorbidity status were not significant predictors in the model. These findings are summarized in Table 5.

**Table 5:** Multivariate logistic regression for predictors of postoperative complications

Variable	Odds ratio (OR)	95% CI	p-value
Hypoalbuminemia	12.68	3.00-53.53	0.001
Age	1.00	0.93-1.07	0.948
Comorbidity	1.89	0.45-8.04	0.388
Surgery duration (min)	1.02	1.00-1.04	0.045

Summary of key findings

The key statistical findings from this study are summarized in Table 6. This table consolidates the primary results across all analytical components, including prevalence of complications, duration of hospital stay, and multivariate predictors. Together, the findings highlight the prognostic importance of serum albumin as an independent and significant marker for postoperative risk stratification in abdominal surgery patients.

**Table 6:** Consolidated summary of key statistical findings

Analysis	Group	Result	Test used	Test statistic	p-value
Complication rate by albumin level	<3.5 g/dL	57.8%	Chi-square test	χ² = 12.64	0.0004
	≥3.5 g/dL	12.5%			
Hospital stay duration	<3.5 g/dL	10.1 ± 2.9 days	Mann–Whitney U test	U = 898.5	<0.0001
	≥3.5 g/dL	6.9 ± 2.0 days			
Logistic regression – albumin	—	OR = 12.68	Adjusted logistic regression		0.001
	—	95% CI: 3.01–53.53			
Logistic regression – surgery time	—	OR = 1.02 per min	Adjusted logistic regression		0.045
	—	95% CI: 1.00–1.04			

## Discussion

This prospective observational study establishes a significant relationship between preoperative hypoalbuminemia and the incidence of postoperative complications in patients undergoing abdominal surgery. Our findings revealed that patients with serum albumin levels below 3.5 g/dL had a markedly higher rate of complications, including surgical site infections, anastomotic leaks, wound dehiscence and delayed wound healing. In addition, these patients experienced significantly longer hospital stays, and multivariate analysis confirmed hypoalbuminemia and prolonged surgery duration as independent predictors of adverse outcomes. The biological rationale underpinning this association is well recognized. Albumin, a negative acute-phase reactant, plays a crucial role in maintaining colloid oncotic pressure, facilitating nutrient transport, modulating inflammation, and supporting tissue repair. Surgical trauma induces systemic inflammation, increasing vascular permeability and leading to a redistribution of albumin into the extravascular space. This phenomenon has been described in several studies, where the immediate postoperative drop in albumin levels (delta albumin) correlates with the intensity of the surgical stress response and clinical outcomes [9]. Our findings are aligned with multiple large-scale studies and meta-analyses that report hypoalbuminemia as a robust predictor of postoperative morbidity and mortality. Labgaa et al. found that a postoperative albumin drop of ≥10 g/L was associated with a threefold increased risk of complications [9]. Similarly, Lalhruaizela et al. showed that preoperative albumin <3.0 g/dL was significantly associated with surgical site infections and wound dehiscence [10]. The logistic regression model further confirmed hypoalbuminemia as an independent and statistically significant predictor of postoperative complications (OR = 12.68; 95% CI: 3.01-53.53; p = 0.001), even after adjusting for age, comorbidity status, and duration of surgery. Notably, surgery duration was also significant (p = 0.045), whereas neither age nor the presence of comorbidities showed independent associations. This highlights that hypoalbuminemia may integrate the impact of several predisposing clinical conditions into a single, measurable risk marker. The strong adjusted odds ratio underlines the prognostic importance of albumin, not just as a reflection of nutritional status but as a robust tool for perioperative risk modeling. The clinical relevance of hypoalbuminemia extends beyond nutritional assessment. Several studies now support its role as a surrogate marker of systemic inflammation and physiological stress [11,12]. Notably, Ge et al. demonstrated that a delta albumin of ≥15% postoperatively predicted major complications with higher sensitivity than CRP alone [11,12]. Our findings reinforce this viewpoint, suggesting that serum albumin-both preoperative and perioperative-can be a powerful, low-cost biomarker to anticipate adverse surgical outcomes. Importantly, our data indicate that hypoalbuminemic patients had a significantly prolonged hospital stay (mean difference: 3.2 days, p < 0.001), consistent with previous reports [13,14]. Kudsk et al. and Issangya et al. both observed a similar association between low albumin levels and extended resource utilization, including ICU admission and delayed return to oral intake [13,14]. These outcomes not only impact patient recovery but also have substantial implications for healthcare costs and resource planning. Interestingly, age and the presence of comorbidities did not emerge as significant independent predictors in our regression model. This may be attributed to the overriding influence of hypoalbuminemia, which integrates multiple risk factors into a single measurable parameter. Galata et al. have argued that albumin reflects both nutritional reserves and the inflammatory burden, making it a more holistic prognostic marker compared to isolated clinical variables [15]. Nevertheless, this study has limitations. The single-center design and modest sample size may limit the external validity of the results. Furthermore, we did not evaluate dynamic perioperative changes in albumin or include other nutritional markers such as prealbumin or phase angle. Finally, although our model adjusts for key confounders, unmeasured variables such as intraoperative fluid shifts or immunosuppressive states could influence outcomes. Future multicenter trials are needed to explore whether targeted interventions such as preoperative nutritional optimization or albumin supplementation can mitigate the risk in hypoalbuminemic patients. Incorporating serum albumin into risk prediction algorithms and perioperative care bundles may also improve patient triage and outcome forecasting.

## Conclusions

This prospective observational study clearly demonstrates that preoperative hypoalbuminemia is a significant and independent predictor of postoperative complications and extended hospital stays in patients undergoing abdominal surgery. Serum albumin serves not only as an indicator of nutritional status but also as a robust biomarker for systemic inflammation and surgical stress response. Routine preoperative assessment of serum albumin levels should be integrated into perioperative protocols to better stratify risk, inform clinical decision-making, and facilitate targeted nutritional and medical interventions, ultimately enhancing patient outcomes and optimizing healthcare resource utilization.
